# Automatic anatomical classification of colonoscopic images using deep convolutional neural networks

**DOI:** 10.1093/gastro/goaa078

**Published:** 2020-12-07

**Authors:** Hiroaki Saito, Tetsuya Tanimoto, Tsuyoshi Ozawa, Soichiro Ishihara, Mitsuhiro Fujishiro, Satoki Shichijo, Dai Hirasawa, Tomoki Matsuda, Yuma Endo, Tomohiro Tada

**Affiliations:** 1 Department of Gastroenterology, Sendai Kousei Hospital, Miyagi, Japan; 2 Department of Internal Medicine, Navitas Clinic, Tokyo, Japan; 3 Tada Tomohiro Institute of Gastroenterology and Proctology, Saitama, Japan; 4 Department of Surgery, Teikyo University School of Medicine, Tokyo, Japan; 5 Department of Surgical Oncology, Graduate School of Medicine, The University of Tokyo, Tokyo, Japan; 6 Department of Gastroenterology and Hepatology, Nagoya University Graduate School of Medicine, Aichi, Japan; 7 Department of Gastrointestinal Oncology, Osaka International Cancer Institute, Osaka, Japan; 8 AI Medical Service, Inc., Tokyo, Japan

**Keywords:** colonoscopy, deep learning, endoscopy, neural network

## Abstract

**Background:**

A colonoscopy can detect colorectal diseases, including cancers, polyps, and inflammatory bowel diseases. A computer-aided diagnosis (CAD) system using deep convolutional neural networks (CNNs) that can recognize anatomical locations during a colonoscopy could efficiently assist practitioners. We aimed to construct a CAD system using a CNN to distinguish colorectal images from parts of the cecum, ascending colon, transverse colon, descending colon, sigmoid colon, and rectum.

**Method:**

We constructed a CNN by training of 9,995 colonoscopy images and tested its performance by 5,121 independent colonoscopy images that were categorized according to seven anatomical locations: the terminal ileum, the cecum, ascending colon to transverse colon, descending colon to sigmoid colon, the rectum, the anus, and indistinguishable parts. We examined images taken during total colonoscopy performed between January 2017 and November 2017 at a single center. We evaluated the concordance between the diagnosis by endoscopists and those by the CNN. The main outcomes of the study were the sensitivity and specificity of the CNN for the anatomical categorization of colonoscopy images.

**Results:**

The constructed CNN recognized anatomical locations of colonoscopy images with the following areas under the curves: 0.979 for the terminal ileum; 0.940 for the cecum; 0.875 for ascending colon to transverse colon; 0.846 for descending colon to sigmoid colon; 0.835 for the rectum; and 0.992 for the anus. During the test process, the CNN system correctly recognized 66.6% of images.

**Conclusion:**

We constructed the new CNN system with clinically relevant performance for recognizing anatomical locations of colonoscopy images, which is the first step in constructing a CAD system that will support us during colonoscopy and provide an assurance of the quality of the colonoscopy procedure.

## Introduction

Total colonoscopy can detect colorectal diseases with high sensitivity and specificity, including colorectal cancer (CRC), colorectal polyps, and inflammatory bowel diseases. The clinical characteristics of colorectal diseases differ among anatomical locations. For example, several recent studies noted differences between right-sided and left-sided CRCs, based on epidemiology, prognoses, and clinical outcomes of chemotherapy [1–5]. Similarly, anatomical locations are essential for the treatment of ulcerative colitis, because the preference for oral medication or suppositories is based on the extent of the colitis. Thus, colonoscopy examinations that can precisely specify the anatomical locations of colorectal diseases are clinically meaningful.

Whereas colonoscopy is commonly used for the screening of positive fecal occult blood tests or abdominal symptoms, the procedure requires specialized training that sufficiently enables practitioners to handle a colonoscope freely, recognize an abnormal region, and diagnose diseases accurately [6]. The first step in adequately examining a colonoscopy is to recognize the anatomical location of each intestinal area during the procedure. Recent evidence suggests that ≥200 cases are required to obtain sufficient colonoscopy-exam skills [6].

Recent remarkable advances in image recognition by computer-aided diagnosis (CAD) systems have made significant impacts in the field of imaging technology and have been applied to various medical areas. Of recent note, the technology of a deep convolutional neural network (CNN) has played an essential role in improving CAD image recognition [7, 8]. Previous research has established that CNNs can achieve clinically useful performance based on the accurate diagnostic ability of many medical areas, such as radiology [9, 10], ophthalmology [11], and pathology [12]. Furthermore, regarding the endoscopic diagnoses, the CAD with CNN detected colon polyps with a sensitivity of >90% [13], gastric cancers with a high sensitivity of 92.2% [14], and *Helicobacter pylori* gastritis with high accuracy [15]. However, no previous study using CNN has investigated the recognition of anatomical locations of colonoscopy, which could be useful to ensure the quality of the procedure.

In the present study, we aimed to construct a CAD system using a CNN to distinguish colorectal images from parts of the cecum, ascending colon, transverse colon, descending colon, sigmoid colon, and rectum. We built a CNN algorithm using thousands of independent colonoscopy images as a development data set and tested its performance using independent colonoscopy images.

## Methods

### Total colonoscopy procedure

We examined images taken during total colonoscopies performed between January 2017 and November 2017 separately at Tada Tomohiro Institute of Gastroenterology and Proctology, Saitama, Japan. The reasons for performing the colonoscopies included abdominal pain, diarrhea, positive fecal immunochemical tests, follow-ups for past colonoscopies at the same center, and simple screening. To correctly identify colorectal anatomical locations, we included images of normal colorectum, which were sufficiently insufflated. Images with abnormal obstacles such as colorectal polyps, cancers, biopsy forceps, and those with severe inflammation, bleeding, and a lot of stools or liquid were excluded. Only white-light or enhanced images with normal magnification were included. The colonoscopy procedure was performed using a standard colonoscope (EVIS LUCERA and CF TYPE H260AL/I, PCF TYPE Q260AI, Q260AZI, H290I, and H290ZI; Olympus Medical Systems, Co., Ltd, Tokyo, Japan) and a colonoscopy system (EVIS LUCERA ELITE; Olympus Medical System, Co., Ltd, Tokyo, Japan). An average of 24 images were taken for each case of the terminal ileum, the cecum, the ascending colon, the transverse colon, the descending colon, the sigmoid colon, the rectum, and the anus during colonoscopy. To train the CNN, the patient information accompanying the images was anonymized prior to algorithm development. We adopted an opt-out approach for patient consent, because this was a retrospective study using anonymized data. The Institutional Review Board of the Japan Medical Association approved this study (ID JMA-IIA00283).

### Images for training and test

**Figure 1.**
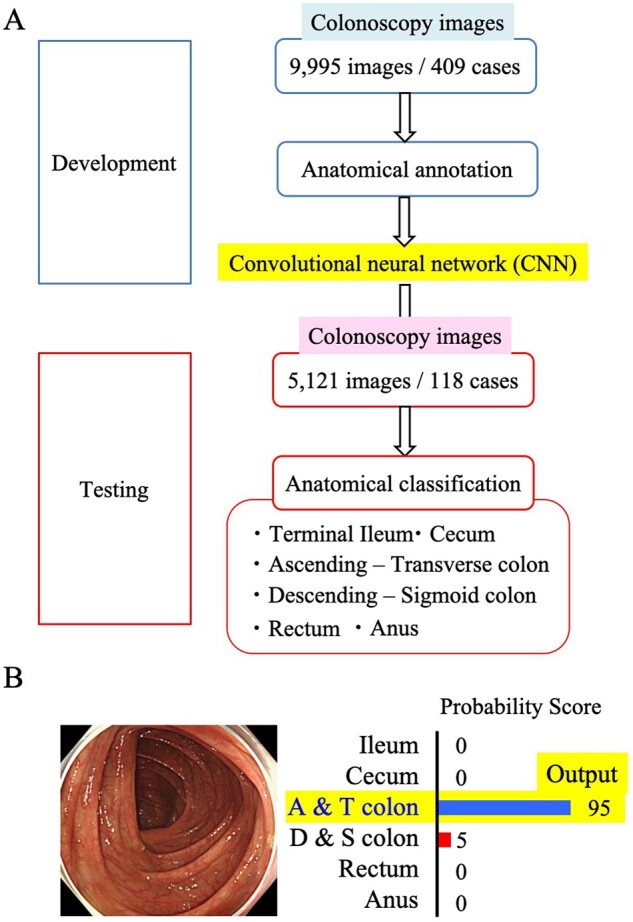
Study design. (A) Flow of the study. The CNN was built by using 9,995 images from 409 cases after anatomical annotation by endoscopists. The constructed CNN classified 5,121 colonoscopy images into six categories; (B) anatomical category that obtained the highest probability score assigned as the category of the image.

**Figure 1** shows the flowchart of the study design. We prepared a development data set for CNN training and validation, which included images for training and validation at a ratio of 80:20. We prepared an independent test data set from other patients. All images from a patient were kept in the same data set. We categorized the images to train the CNN into seven categories: the terminal ileum, the cecum, the ascending colon to transverse colon, the descending colon to sigmoid colon, the rectum, the anus, and indistinguishable parts. By arranging images of each case in order of the time that the images were taken, we categorized serial images based on the characteristic parts, such as the hepatic flexure, splenic flexure, and curve of the sigmoid descending junction. All images for training were checked for categorization by two doctors (Ozawa, Tada) prior to CNN training. Tada is a certified endoscopist from the Japan Gastroenterological Endoscopy Society with >10 years’ experience of endoscopy and Ozawa has participated in >500 colonoscopy procedures for >10 years with the equivalent endoscopic experience to that of a certified endoscopist. Their discussions were used to resolve cases of disagreement.

**Figure 2.**
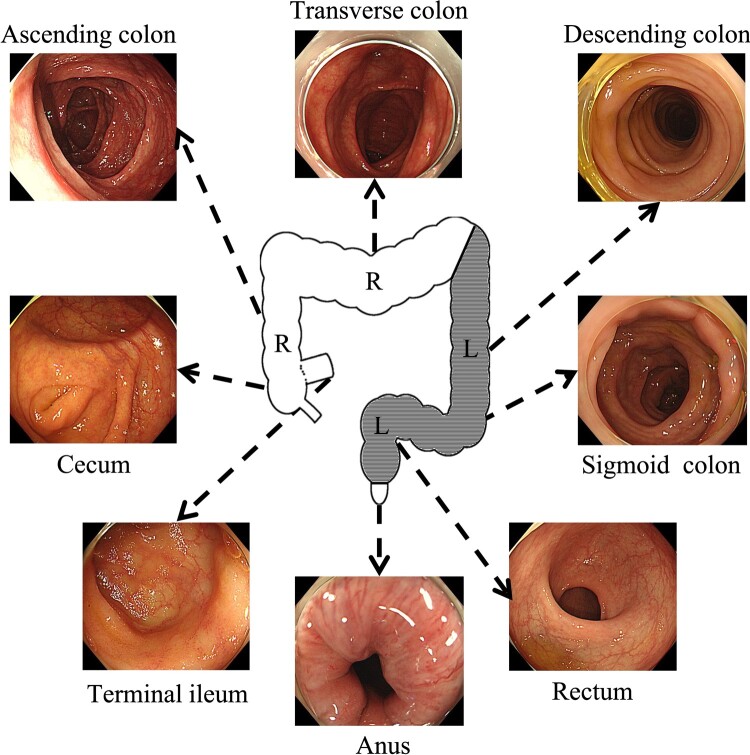
Anatomical category and subcategory. The cecum, ascending colon, and transverse colon were collectively defined as the right-sided colon (R), whereas the descending colon, sigmoid colon, and rectum were defined as the left-sided colon (L).

Test-set images were divided into six categories: the terminal ileum, the cecum, the ascending colon to transverse colon, the descending colon to sigmoid colon, the rectum, and the anus. Indistinguishable images were not included in the test set. We integrated the category of the cecum and the ascending colon to the transverse colon as the ‘right-sided colon’ and that of the descending colon to the sigmoid colon and the rectum as the ‘left-sided colon’ (**Figure 2**).

We estimated the number of images required to construct a CNN based on several previous studies that addressed the effects of CADs for colorectal polyps by learning ≤5,000 images [7, 13, 16]. Therefore, we aimed to construct our CNN system based on ∼10,000 images to ensure a sufficient amount of data. In total, 9,995 images of 409 cases collected were prepared for the development image set and we used 5,121 images of 118 cases for the test image set. The numbers of images for each anatomical category of both image sets are shown in **Supplementary Table 1**.

### CNN algorithms

We constructed our system with a deep CNN, GoogLeNet, without altering its original algorithm. GoogLeNet is an expressive neural network with sufficient parameters, consisting of 22 layers of deep network. We used the Caffe deep-learning framework, provided by the Berkeley Vision and Learning Center [17], to train and test the CNN. All the layers of the CNN were fine-tuned from ImageNet [18] data with AdaDelta [19] using a learning rate of 0.005 and a batch size of 32. Each image was resized to 224 × 224. To acquire a high-performance CNN model, we had to identify appropriate values for hyper-parameters such as learning rate with repeated trial and error. For calculations, we used Intel Core i7-7700 K as the central processing unit and GetForce GTX 1070 as a graphics-processing unit.

For images of the independent test data set, the CNN system provided a probability score (PS) ranging from 0 to 1, representing the probability of an image belonging to a particular category. The CNN calculated the PS for the seven categories on each image. The anatomical category that obtained the highest PS was assigned as the category of the image (**Figure 1B**).

### Analyses

The main outcomes of the study were the sensitivity and specificity of the CNN for the anatomical categorization of colonoscopy images. The receiver-operating characteristic (ROC) curves were drawn for each category and the areas under the curves (AUCs) were calculated by GraphPad Prism 7 (GraphPad software, Inc., California, USA). We also calculated the sensitivity and specificity for particular PSs of >90%, 80%, 70%, and 60%.

## Results

The newly constructed CNN system correctly recognized 66.6% (3,410/5,121) of the test-set images. The CNN categorized each image for 0.00175 s. **Table 1** shows the rates of correct recognition with a PS provided by the CNN. It provided 9.9% (507/5,121) of images with a PS of >99%, showing an accuracy of 91.7%. However, 7.3% (372/5,121) of images with a PS of <50% showed an accuracy of 36.6%.

**Table 1.** Distribution of the probability score and CNN accuracy

Probability score	Correct (%)	Whole (%)	Accuracy (%)
>99 %	465 (14)	507 (10)	91.7
90%<, equal to or less than 99%	1,039 (30)	1,296 (25)	80.2
70%<, equal to or less than 90%	1,009 (30)	1,549 (30)	65.1
50%<, equal to or less than 70%	761 (22)	1,397 (27)	54.5
Equal to or less than50%	136 (4)	372 (7)	36.6
Total	3,410 (100)	5,121 (100)	66.6

All values are presented as numbers of cases followed by percentages in parentheses.

**Figure 3.**
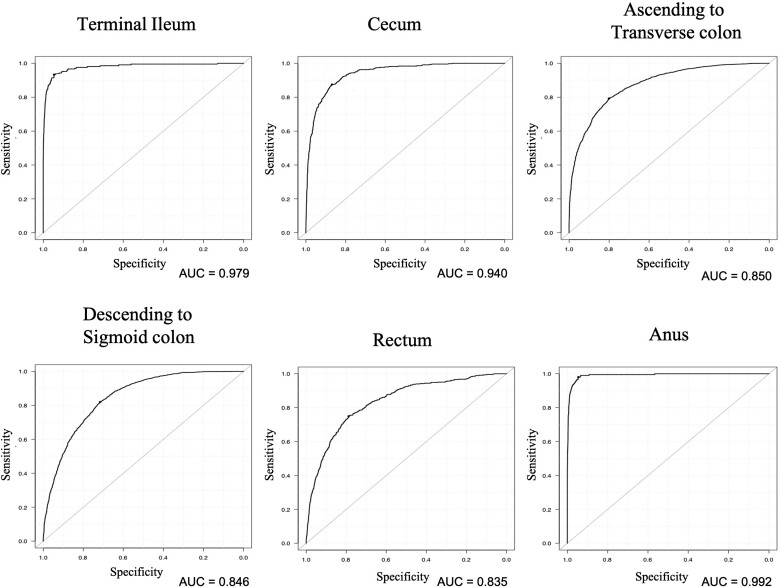
The CNN recognized the anatomical location of CS images with an AUC of 0.979 for the terminal ileum, 0.940 for the cecum, 0.850 for ascending colon to transverse colon, 0.846 for descending colon to sigmoid colon, 0.835 for the rectum, and 0.992 for the anus.

**Table 2** shows the distribution of the output of the CNN according to each anatomical category. The CNN recognized images of the anus with the highest sensitivity (91.4%), whereas it recognized images of the rectum with the lowest sensitivity (23.3%). The specificity of each anatomical category was >90%, except for the descending colon to sigmoid colon categories (60.9%). The ROCs for all categories are shown in **Figure 3**. The CNN recognized the images with AUCs of >0.8 for each anatomical location. **Table 3** shows the distribution of the CNN output in five categories: the terminal ileum, the right-sided colon, the left-sided colon, the anus, and indistinguishable parts. The category of the left-sided colon indicated high sensitivity of 91.2% and relatively low specificity of 63.7%, whereas the categories of terminal ileum, right-sided colon, and anus showed the opposite outcome. **Table 4** shows a sensitivity and specificity per the PS. For all categories except for the rectum, the higher the PS, the higher the sensitivity and specificity. Additionally, the sensitivity of the rectum was inconsistent with the PS.

**Table 2.** Anatomical classification of CS images into seven CNN categories

Anatomical categories	Terminal ileum	Cecum	Ascending colon to transverse colon	Descending colon to sigmoid colon	Rectum	Anus
	(*n* = 209)	(*n* = 423)	(*n* = 1,742)	(*n* = 2,081)	(*n* = 467)	(*n* = 199)
CNN output^a^						
Terminal ileum	145 (69)	13 (3)	4 (0)	11 (1)	6 (1)	0 (0)
Cecum	9 (4)	211 (50)	64 (4)	7 (0)	4 (1)	0 (0)
Ascending colon to transverse colon	6 (3)	89 (21)	891 (51)	108 (5)	6 (1)	1 (1)
Descending colon to sigmoid colon	40 (19)	97 (23)	775 (44)	1,872 (90)	265 (57)	13 (7)
Rectum	1 (0)	4 (1)	1 (0)	78 (4)	109 (23)	3 (2)
Anus	8 (4)	9 (2)	7 (0)	5 (0)	77 (16)	182 (91)
Indistinguishable parts	0 (0)	0 (0)	0 (0)	0 (0)	0 (0)	0 (0)
Sensitivity (%)	69.4	49.8	51.1	90.0	23.3	91.4
Specificity (%)	99.3	98.2	93.8	60.9	98.1	97.8

a All values are presented as numbers of cases followed by percentages in parentheses.

**Table 3.** Anatomical classification of CS images into five CNN categories

Anatomical categories	Terminal ileum	Right-sided colon	Left-sided colon	Anus
	(*n* = 209)	(*n* = 2,165)	(*n* = 2,548)	(*n* = 199)
CNN output^a^				
Terminal ileum	145 (69)	17 (1)	17 (1)	0 (0)
Right-sided colon	15 (7)	1,255 (58)	125 (5)	1 (1)
Left-sided colon	41 (20)	877 (41)	2,324 (91)	16 (8)
Anus	8 (4)	16 (1)	82 (3)	182 (91)
Indistinguishable parts	0 (0)	0 (0)	0 (0)	0 (0)
Sensitivity (%)	69.4	58.0	91.2	91.5
Specificity (%)	99.3	95.2	63.7	97.8

a All values are presented as numbers of cases followed by percentages in parentheses.

**Table 4.** Sensitivity and specificity according to probability score (PS)

Anatomical category	Terminal ileum	Cecum	Ascending colon to transverse colon	Descending colon to sigmoid colon	Rectum	Anus
PS >60						
Sensitivity (%)	80.1	62.7	52.5	94.7	18.1	94.1
Specificity (%)	99.6	98.9	97.0	61.6	98.9	98.0
PS >70						
Sensitivity (%)	81.8	67.6	53.6	96.2	15.1	95.1
Specificity (%)	99.7	99.0	98.0	63.0	99.1	97.9
PS >80						
Sensitivity (%)	88.2	77.0	55.6	97.6	12.4	96.6
Specificity (%)	99.8	99.2	99.0	66.8	99.5	97.9
PS >90						
Sensitivity (%)	92.2	82.7	56.9	99.1	8.2	97.0
Specificity (%)	99.8	99.3	99.5	72.9	99.9	97.5

We reviewed 1,711 images that were incorrectly categorized by the CNN to improve the performance of the CNN in further study. The CNN system provided 17.5% (299/1,711) of incorrectly recognized images with a PS of >0.9.

**Figure 4.**
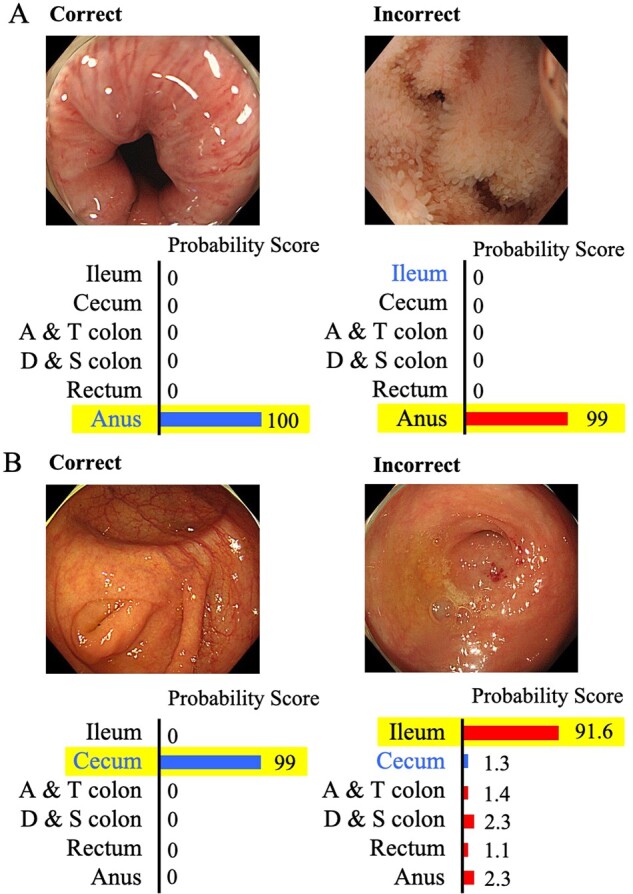
Correctly and incorrectly recognized images by the CNN. (A) The CNN incorrectly recognized an image of the terminal ileum as the anus. The outline of the lumen was similar to that of the anus; (B) the CNN incorrectly recognized an image of the cecum as a terminal ileum in which an appendix hole could be seen as one of the features of the cecum.

**Figure 4** illustrates the typical cases incorrectly recognized by the CNN.

## Discussion

We constructed our CNN system based on 9,995 colonoscopy images of 409 cases and showed its clinically relevant performance to identify the anatomical locations using a sizeable independent test data set. The CNN system recognized the images of the colon with an accuracy rate of >60%. Moreover, because the accuracy rate was high enough for the terminal ileum, we will be able to construct an automatic confirmation system for the completion of the total colonoscopy if we incorporate it into a real-time colonoscopy procedure. Thus, we could construct the basis for the development of future colonoscopies using a CAD system.

In the present study, the accuracy differed by the PS. Generally, because images with high probability scores are recognized with high accuracy, the CNN can perform better by being limited to only images with high probability scores. When limited to probability scores of >99%, the accuracy rate was >90%, which may be clinically practicable. However, when we only consider the images with an accuracy of >90%, we exclude other correctly categorized images that account for >80% of whole images. We should elucidate the way in which the CNN can provide more images with high probability scores and validate the adequate values of probability scores in further studies.

The anatomical recognition may be more difficult in colonoscopy than in gastrointestinal endoscopy. As previously reported, the constructed CNN could categorize gastrointestinal images with high sensitivity and specificity: 93.9% and 100% for the larynx, 95.8% and 99.7% for the esophagus, 98.9% and 93.0% for the stomach, and 87.0% and 99.2% for the duodenum [20]. Clinicians can usually recognize where colonoscopy images are during clinical practice by considering their sequential order or their relationship with former or consecutive images. Therefore, the accuracy recognition rate of 66%, based on single images for the present CNN, could be improved if the relationship between the former and latter images was integrated in the future version of the algorithm.

The sensitivity and specificity of the CNN system differed according to the anatomical categories. The category of descending colon to sigmoid colon had a sensitivity of >90%, whereas it had its lowest specificity at 60.9%. By contrast, the categories of the terminal ileum, the cecum, ascending colon to transverse colon, and the rectum had high specificities and low sensitivities of 23.3%–69.4%. The system recognized the anus with high sensitivity and a specificity of >90%, which may be due to characteristic shapes of the anus. Interestingly, the CNN provided incorrect output for rectal images with certainty and mostly recognized rectal images as descending colon to sigmoid colon. The lack of characteristic parts in the rectum may lead to low sensitivity. However, the CNN showed relatively low sensitivity for the terminal ileum or the cecum categories, whereas both had characteristic parts, such as the villi of ileum, the ileocecal valve, and the appendix orifice. As previously reported, the appendix orifice can be automatically detected by characterizing the appendix [21, 22]. As shown in the recent research that required >5,000 images of the caecum, a sufficient number of a particular part enabled the CNN to recognize it easily [23]. For teaching the CNN system, increasing the number of images of each characteristic part is one of the ways in which to improve the accuracy to identify it, which may make the detection rate of the anatomical category more accurate.

Furthermore, to enhance the precise locating ability, combining colonoscopy with other modalities that show 3D information, such as computer tomography and fluoroscopic images, may be useful. Using colonoscopy videos is also a further research step. Recently, Yu *et al.* [24] reported a polyp-detection system in colonoscopy videos by combining a 2D CNN with a 3D fully convolutional network, which performed better than previous methods. These further technical applications involving more spatial information may be effective for constructing a CAD with anatomical-recognition ability.

For medical-image recognition with CAD, there have been several machine-learning systems such as CNN and a support vector machine (SVM). Although it remains unclear which system is better at recognizing the anatomical location of colonoscopy images, recent research dedicated to detecting colon polyps reported that systems using SVM as a classification and combined with CNN have good results [25]. The type of CNN used is also important. Among several deep CNNs, we adopted a well-known CNN, GoogLeNet [26], as one of the benchmarks. It would be difficult to suppress overfitting during CNN learning from our data set if a larger CNN, such as ResNet [27], was used. In a future study, comparing the outcomes of machine-learning systems is necessary.

The capability to automatically recognize the anatomical location of the colon would have a substantial impact on both diagnosis and treatment. First, the detection of the terminal ileum could provide us with an assurance of completion of the total CS, and we will objectively be able to calculate the withdrawal from the time of the first detection of the terminal ileum to that of last detection of the anus. Both the caecal intubation rate and the withdrawal time are considered the key performance indicators of colonoscopy [28]. A lower caecal intubation rate is a risk factor for post-colonoscopy CRC and sufficient withdrawal time is necessary for a high adenoma-detection rate [29]. Furthermore, the withdrawal time for each anatomical part may be associated with the polyp-detection rate. The endoscopists should take sufficient time to observe the transverse and sigmoid colons because polyps in the transverse or sigmoid colons are likely to be missed with short withdrawal time [30]. With the assurance of the total colonoscopy and a measure of withdrawal time, a CNN with the capability of anatomical recognition will facilitate an accurate colonoscopy procedure. Third, we will be able to automatically recognize the affected locations by colonic diseases, such as inflammatory bowel diseases, segmental infectious or ischemic colitis, diverticulosis, with the help of the CAD system more efficiently. For example, for the classification of ulcerative colitis, we generally choose a treatment or drug regime based on the extent of the colitis. Likewise, regarding CRC, the anatomical location of the disease is essential for surgery.

Furthermore, the automatic-recognition system for anatomical information would be useful for trainees. To achieve competency at colonoscopy, or a caecal intubation rate of 90%, trainees require >200 colonoscopies with high intensity training [31]. One of the most challenging factors for trainees to achieve successful insertion and observe during withdrawal is to realize where the scope is. The anatomical-recognition function may help them to recognize the location of the scope, which may help improve to the efficiency of their training.

There are several limitations to consider. First, the accuracy rate depended on the ability or skills of the person who categorized the test images. The test data set that we prepared may have contained some misclassified images. Second, all images prepared in the present study were obtained from a single institute. The number of colonoscopy images per location, the use of or the kinds of caps, the degree of air distention, and the use of water infusion can differ because of the practitioner’s or the institute’s policy. Thus, it is unclear whether our CNN system could work in the same way for images taken from other institutes. Third, images with abnormalities were excluded. To develop CNNs to work correctly in a clinical setting, the system must classify images with abnormalities, such as feces, bubbles, and polyps, in the future. Finally, although we used more images for both development and test data sets than used by any other study on colonoscopy CNNs, we may require even more images to construct a more reliable CNN. The imbalances in the numbers of images between anatomical locations must be resolved.

## Conclusion

The present study revealed the clinically relevant performance of the new CNN system in view of the anatomical location of colonoscopy images. This is the first step towards constructing a CAD system to provide support during colonoscopy and ensure the quality of the colonoscopy procedure. Further prospective multi-institutional research would be required to assess the ability of our CAD system more rigorously.

## Supplementary data

Supplementary data is available at *Gastroenterology Report* online

## Authors’ contributions

H.S., To.T., and S.S. conceived and designed this study. Y.E. and To.T. performed the acquisition of data; H.S., T.O., S.I., and M.F. analysed and made interpretation of the data; H.S., To.T., Te.T., and T.O made drafts of the manuscript, All authors made critical revision for intellectual content. All authors read and confirmed the final version of the manuscript.

## Funding

This work was not supported by any funding.

## Supplementary Material

goaa078_Supplementary_Data
