# Supplementary material for: Automatic anatomical classification of colonoscopic images using deep convolutional neural networks
Source: Gastroenterol Rep (Oxf). 2020 Dec 7;9(3):226–33. doi: 10.1093/gastro/goaa078 (PMC8309686; doi:10.1093/gastro/goaa078)
Supplement: goaa078_Supplementary_Data [file goaa078_supplementary_data.docx]

**Supplementary Table 1. Anatomical classification of the training and validation image set**

| Category | Sub-category | No. of training set (%) | No. of validation set (%) |
| --- | --- | --- | --- |
| Terminal ileum |  | 652 (6.5) | 209 (4.1) |
| Cecum | Right-sided | 1,048 (10.5) | 423 (8.3) |
| Ascending, Transverse |  | 2,376 (23.8) | 1,742 (34.0) |
| Descending, Sigmoid | Left-sided | 3,535 (35.4) | 2081 (40.6) |
| Rectum |  | 1,037 (10.4) | 467 (9.1) |
| Anus |  | 970 (9.7) | 199 (3.9) |
| Indistinguishable |  | 377 (3.8) | 0 (0) |
| Total |  | 9,995 (100) | 5,121 (100) |
